# From data to drug: the translational impact of RaDaR, the UK national registry of rare kidney diseases

**DOI:** 10.1093/ndt/gfaf227

**Published:** 2025-10-25

**Authors:** Daniel P Gale

**Affiliations:** UCL Department of Renal Medicine, Royal Free Hospital, London, UK; National Registry of Rare Kidney Diseases (RaDaR), UK Kidney Association, Bristol, UK

**Keywords:** clinical trials, patient registry, rare kidney disease, real-world data, surrogate endpoints

## Abstract

Each rare kidney disease affects relatively few individuals, but collectively these disorders place a significant burden on patients, families, healthcare systems and society. Historically, therapeutic development has been limited by diagnostic challenges, small and underpowered trials, and a lack of disease-specific, clinically meaningful endpoints acceptable to regulators. The UK National Registry of Rare Kidney Diseases (RaDaR) was launched in 2010 to address these challenges by capturing high-quality longitudinal data. With over 37 000 participants enrolled across more than 100 UK sites, RaDaR now functions as a nationally integrated research platform supporting epidemiological studies, biomarker and genomic research, clinical trial feasibility and recruitment, patient-reported experience studies, and health economic evaluations. RaDaR’s infrastructure and stakeholder engagement have advanced understanding of disease natural history and surrogate endpoints, and have informed trial design. Analyses from RaDaR have helped quantify progression risk in immunoglobulin A nephropathy and other glomerulopathies, supporting proteinuria reduction as a surrogate endpoint for regulatory approval. RaDaR data have informed National Institute for Health and Care Excellence (NICE) appraisals and supported clinical trials targeting specific molecular subgroups. By integrating clinical, laboratory and hospital episode data, and by maintaining sustained patient engagement, RaDaR enables research that is both scalable and patient-centred. Its role in trials for focal segmental glomerulosclerosis, Alport syndrome and C3 glomerulopathy exemplifies its contribution to precision nephrology. For the rarest conditions, international collaboration remains essential to achieve adequately powered datasets and harmonized endpoints. RaDaR demonstrates how a national registry can bridge the gap between real-world data and therapeutic development, accelerating the path from disease understanding to drug approval in rare kidney disease.

## THE RARE DISEASE PROBLEM

**Figure 1:**
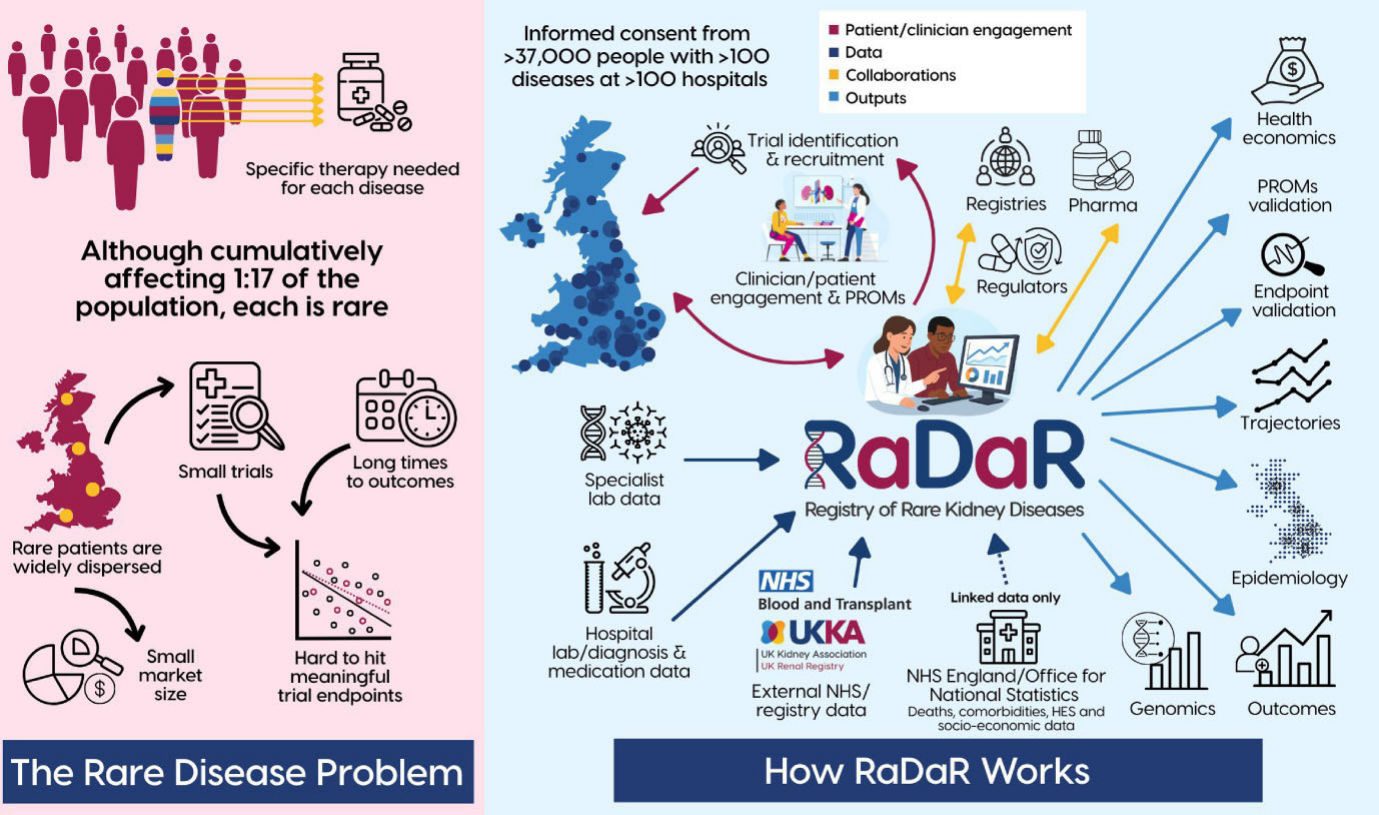
The rare disease problem (left) and how RaDaR works (right). PROMs, patient-reported outcome measures [6, 7, 9, 13].

Rare diseases, typically defined as affecting less than around two to ten people per 10 000 population (depending on criteria used), collectively affect a substantial proportion of the population. By some estimates as many as 1 in 17 people will be affected by a rare disease during their lifetime. Despite this significant burden on patients, their families, healthcare systems and wider society, therapeutic development has lagged behind advancements for more common conditions. There are several factors (summarized in Fig. 1, left panel) contributing to this therapeutic gap:

- Limited awareness and diagnostic infrastructure: the severity and poor clinical outcomes of many rare diseases remain under-recognized by the broader medical and regulatory community. In some cases, appropriate diagnostic tests are not widely available or routinely used. This lack of visibility can hinder the ability of regulatory agencies and healthcare providers to appropriately weigh the potential benefits of a novel treatment against its known and theoretical risks and cost.
- Early-onset and progressive nature of disease: many rare diseases, particularly monogenic ones, present in childhood and progress over decades. Consequently, interventions must be initiated early to meaningfully alter outcomes. For example, in Alport syndrome, trials may need to be conducted in children to prevent long-term kidney damage, even though kidney failure often occurs in adulthood. This introduces additional ethical and regulatory considerations that can complicate or delay trial enrolment in paediatric populations.
- Need for disease-specific therapeutics: in serious rare diseases with a high likelihood of serious illness or death, disease mechanisms are unlikely to be shared with more common disorders. For example, it is unlikely that therapies effective in slowing chronic kidney disease (CKD) progression in common conditions such as diabetes or cardiovascular disease will be sufficient to prevent kidney failure. Each rare disease will likely require a therapy specifically tailored to its underlying pathophysiology (and in some cases, to specific molecular subtypes) to meaningfully alter the disease course and prevent (rather than merely delay) serious illness.
- Challenges in trial design: the rarity of individual diseases limits the number of patients available for clinical trials. Additionally, definitive clinical outcomes—such as kidney failure or mortality—may occur years after disease onset. As a result, most trials are underpowered to detect effects on hard outcomes within feasible timeframes, complicating regulatory interpretation.
- Uncertain variability of surrogate endpoints: surrogate markers like proteinuria and estimated glomerular filtration rate (eGFR) are often used in trials for rare kidney diseases, but the expected variability in these measures—especially in children or in conditions such as nephrotic syndrome—is not well established. This hampers power calculations and limits confidence in trial design and interpretation.
- Feasibility of patient recruitment: eligibility criteria based on genotype or phenotype may define populations so narrowly that recruitment becomes unfeasible. Trials can fail simply because eligible patients are too few or too difficult to identify within a realistic timeframe.
- Geographic dispersion of patients: patients with rare diseases are often widely dispersed across countries or regions. This necessitates the opening of many trial sites to meet recruitment targets, which increases cost and complexity. Moreover, patients not engaged with research-active centres may have limited access to potentially transformative therapies under investigation.
- Small potential market size: because the number of patients affected by each rare disease is small, the opportunities for recovery of the investment needed to develop and test therapies are limited. This increases the perceived risk, on the part of both commercial and academic funders, of investing in this area and can have the downstream consequence that where effective therapies are developed their cost is so great that not all patients are able to access them.

In nephrology, these issues are particularly relevant: analysis of registry data, such as the UK Renal Registry, indicates that while rare kidney diseases account for only a small proportion of all individuals with CKD, they are disproportionately represented among patients with kidney failure. In the UK, glomerulonephritis is the single most common diagnostic category among prevalent recipients of renal replacement therapy, ahead of diabetes, hypertension and renovascular disease [1]. Among children, the contribution of rare kidney diseases is even more striking, comprising the vast majority of those living with kidney failure [2]. To stretch the Anna Karenina principle, healthy kidneys are all alike, but each unhealthy kidney is unhealthy in its own way.

To begin addressing some of the systemic challenges facing research in rare kidney diseases, the Renal Association (now the UK Kidney Association, UKKA) in partnership with the British Association for Paediatric Nephrology launched a national rare disease strategy in 2010 [3, 4]. A cornerstone of this initiative was the creation of the National Registry of Rare Kidney Diseases (RaDaR), alongside coordinated efforts to build communities of patients, clinicians and researchers to share data, expertise and support. The goal was to catalyse improvements in care and accelerate the development of evidence-based treatments for rare renal conditions. Funding was initially provided by Kidney Research UK, Kidney Care UK and the UK Medical Research Council, with ongoing financial and infrastructure support maintained by the UKKA.

Creating a multi-disease national registry inevitably brings significant operational, legal and organizational challenges. Many of these were addressed—though not always immediately—by drawing on the data-handling, governance and informatics expertise of the UK Renal Registry (also maintained by the UKKA) and the wider renal, healthcare and scientific community. For rare kidney diseases, specific obstacles included the need to engage patients and carers across many different conditions and geographic locations, as well as uniting a diverse community of nephrologists, scientists and allied professionals under a single national initiative. From the outset, the approach taken prioritized inclusivity and decentralization—across both patients and professional stakeholders—even at the cost of some uniformity and granularity. Eligibility criteria were deliberately broad, and streamlining recruitment was consistently prioritized over comprehensive baseline data entry to ensure that the registry gained critical mass before focusing on depth.

These strategic choices help explain the long lag-phase between RaDaR’s establishment in 2010 and the emergence of clear translational impact more than a decade later. A key lesson learned has been the importance of patience, iterative development and stakeholder engagement: building a national resource of this scale required time to earn trust, achieve coverage, and develop the infrastructure and analytical capacity needed to gather and transform raw data into meaningful clinical insights.

## HOW RaDaR WORKS

RaDaR is hosted and operationalized within the UKKA which has mature and well-established data security and information governance capabilities integral to the UK Renal Registry. Each participant (or their parent/carer) provides written, informed consent for their past, present and future clinical, research or other healthcare record to be stored and analysed for the purpose of research, and to be contacted about future research opportunities. For many participants, the possibility of participating in research and clinical trials is a key motivation for enrolment.

RaDaR recruitment is supported by its adoption onto the National Institute for Health Research (NIHR) Clinical Research Network Portfolio, which provides per-patient reimbursement to participating National Health Service (NHS) sites. This decentralized funding model has enabled the expansion of research capacity across UK healthcare facilities, including many sites beyond traditional academic centres. Notably, this infrastructure contributed to the rapid scaling of national efforts such as the COVID-19 RECOVERY (Random Evaluation of COVID-19 Therapy) trial which played such a pivotal role informing clinical management globally of COVID-19 [5].

At the point of recruitment, only a minimal core dataset is entered manually by clinical or research staff. This includes diagnosis (as free text SNOMED CT, ICD-10 (International Classification of Diseases, 10th revision) or European Renal Association–European Dialysis and Transplant Association codes) and its date, family history and a few disease-specific variables. This streamlined approach enabled over 25 000 participants to be recruited between 2010 and 2020, with over 37 000 enrolled at the time of writing across its now 33 rare disease group cohorts (Table 1).

**Table 1:** RaDaR metadata showing recruitment and data availability for each RDG cohort as of August 2025.^a^

RDG cohort	Cohort size	Number with lab results	Number receiving RRT	Number with creatinine readings	Mean number of creatinine readings per patient
Alport syndrome	1246	938	440	1056	90
APRT deficiency	10	7	2	9	75
Atypical HUS	341	274	162	283	172
Autosomal dominant polycystic kidney disease	9174	7575	3688	8010	96
Autosomal dominant tubulointerstitial kidney disease	272	215	120	223	98
Autosomal recessive polycystic kidney disease/nephronophthisis	277	225	124	246	120
BK nephropathy	175	164	148	164	361
Calciphylaxis	78	67	63	67	284
CKD due to genetic factors in people of African ancestry	689	252	161	253	146
CMV post-transplant	697	615	676	651	307
Congenital anomalies of the kidneys and urinary tracts	430	261	66	315	76
Cystinosis	200	173	110	178	174
Cystinuria	541	361	10	399	30
Dent disease and Lowe syndrome	82	48	17	53	74
Fabry disease	65	54	27	57	103
Fibromuscular dysplasia	108	65	0	64	15
*HNF1B* mutations	130	91	21	98	50
Hyperoxaluria	145	118	43	122	93
Idiopathic nephrotic syndrome	5085	3781	1205	4313	91
IgA nephropathy	5398	4541	2641	4693	125
Inherited renal cancer syndromes	448	22	4	84	20
Lupus nephritis	273	163	10	177	79
Membranoproliferative glomerulonephritis	1285	1047	643	1189	140
Membranous nephropathy	2994	2291	608	2455	85
Mitochondrial renal disease	6	2	0	2	79
Monoclonal gammopathy of renal significance	273	209	114	213	137
Pregnancy	825	644	257	731	113
Pure red cell aplasia	9	6	6	6	241
Retroperitoneal fibrosis	175	120	22	138	72
STEC-associated HUS	201	121	86	133	57
Tuberous sclerosis	304	213	22	222	44
Tubulopathy	491	313	17	364	43
Vasculitis	5908	4135	1245	4458	91

^a^Latest figures can be found at https://www.ukkidney.org/rare-renal/metadata.

APRT, adenine phosphoribosyltransferase; RRT, renal replacement therapy; HUS, haemolytic uraemic syndrome; STEC, Shiga toxin-producing *Escherichia coli*; CMV, cytomegalovirus.

Each rare kidney disease, or related group of diseases, is represented within RaDaR by a Rare Disease Group (RDG). Each RDG includes at least one nephrologist and one patient representative, alongside other interested clinicians, researchers and healthcare professionals. RDGs are responsible for proposing the relevant disease groups for inclusion and defining the eligibility criteria and data fields to be collected; maintaining patient and clinician-facing pages on the RaDaR website; organizing annual meetings and patient information days; and driving research initiatives using RaDaR data. Having at least one motivated clinician, with enough time to devote the effort required, is therefore needed for each disease to be included in RaDaR.

RaDaR integrates information from multiple data streams (see Fig. 1), including:

- Manual entry of key clinical details at the point of recruitment;
- Automated feeds of laboratory results from hospitals;
- Linkage with the UK Renal Registry, providing dialysis initiation information;
- Linkage with NHS Blood and Transplant, ensuring accurate recording of transplant events;
- Linkages to national datasets, including the Office for National Statistics and NHS England (mortality and socioeconomic data), Hospital Episode Statistics (HES; for emergency and inpatient care, which uses ICD10 codes), and specialist UK laboratories (e.g. genetic, genomic, immunology, histopathology). Genetic test results are stored using either Human Genome Variation Society nomenclature or Variant Call Format, with genome build and/or transcript specified as appropriate, where stated in the report. RaDaR does not currently hold genome-wide variant or sequencing data on its participants.

For selected conditions, dedicated research staff at certain hospitals supplement RaDaR data by manually extracting additional information from medical records—such as kidney ultrasound findings or biopsy results—not otherwise reliably captured through automated sources. Where funding is available, RaDaR or delegated site staff can enrich the data held for specific patient groups—for instance by manually transferring kidney length information from radiology studies performed in patients with autosomal dominant polycystic kidney disease into RaDaR. This allows the granularity of data in the targeted disease cohort to approach that possible in dedicated disease-specific research cohort studies. The large size and long median follow-time of some RaDaR cohorts makes this a potentially powerful mechanism for future research studies.

Through a combination of central coordination, widespread stakeholder (especially patient) engagement, decentralized funding, minimal data burden at entry and scalable digital infrastructure, RaDaR has become a useful research platform able to support not only epidemiology [6] and natural history [7] studies, but also serving as a mechanism for enrolment into national genomic and biomarker research studies (for instance the NIHR BioResource for Rare Diseases [8, 9] and NURTuRE, the National Unified Renal Translational Research Enterprise [10]). Furthermore, it also serves as a national clinical trial readiness platform—accelerating patient identification, feasibility assessments and recruitment for studies in rare kidney disease. This infrastructure is illustrated in the Fig. 1 (right panel) and further operational and governance details, as well as metadata, are available within the RaDaR website (https://rarerenal.org), which is hosted by the UKKA. Initial analyses to 2020 indicate that, among UK patients receiving renal replacement therapy, around 40% of those eligible for inclusion in RaDaR have been recruited and although there was some variability, we did not observe systematic recruitment biases—either by disease, geography or demographic characteristics [6].

## RaDaR’s COLLABORATIVE ANALYTICAL FRAMEWORK

A large repository of data is a powerful asset, but on its own it is insufficient to generate the insights needed to advance patient care. A key challenge for RaDaR has therefore been to develop, in parallel, the informatics infrastructure and statistical expertise required to support robust analysis, and to connect that capability with clinical, patient, academic, regulatory and industry stakeholders in a way that enables meaningful research.

RaDaR’s RDG structure inherently facilitates clinician and patient engagement, embedding real-world expertise and lived experience into the design and interpretation of analyses through leadership and representation within each RDG of a nephrologist and a patient (or representative of a patient charity). However, it was the development of an effective model for industry collaboration that proved particularly catalytic for advancing therapeutic development: commercial partners are ideally placed to identify the key gaps in knowledge holding back drug development. Over time, RaDaR has established a partnership model in which, following submission of an expression of interest via the RaDaR web page (https://rarerenal.org), analyses are carried out by a RaDaR analytical team, typically comprising one or more RDG representatives (including clinicians and patients, where appropriate), a RaDaR statistician and often a renal medicine clinical trainee. This internal team collaborates iteratively with external partners—such as pharmaceutical companies or regulatory agencies—through a process that allows shared analytical goals while preserving data governance and participant privacy. Crucially, while metadata and aggregate results can be freely shared, individual-level participant data remain accessible only to the RaDaR team. This model protects confidentiality of participants’ extremely sensitive genetic, clinical and demographic data; mitigates legal and ethical risks for all parties; and reduces the likelihood of duplicate publications or disproportionate representation of RaDaR data in global analyses of rare diseases. An additional benefit has been the training of future renal clinician scientists who have the opportunity to acquire both the quantitative data skills and specialized clinical expertise that will allow them to drive further clinically focussed rare kidney disease research in the future.

## REAL-WORLD IMPACTS OF REAL-WORLD DATA

In 2023, in a collaboration with Travere Therapeutics, RaDaR reported outcomes among 2439 patients with biopsy-confirmed immunoglobulin A (IgA) nephropathy meeting entry criteria of proteinuria >0.5 g/day or eGFR <60 mL/min/1.73 m² at any time in their disease. This analysis showed that even among individuals with time-averaged proteinuria <0.5 g/day, around 20% progressed to kidney failure within 10 years, with around 30% reaching this endpoint by then in the 0.5–1 g/day group. Crucially, modelling showed that maintaining an eGFR decline rate ≤1 mL/min/1.73 m²/year is necessary in most patients to prevent progression to kidney failure over a lifetime [11]. These findings reframed disease understanding by emphasizing that targeting proteinuria <1 g/day will likely be insufficient to prevent kidney failure in most patients, and that more potent, disease-modifying therapies are needed [12]. This work also provided a template for using registry-based data to define clinically meaningful targets for surrogate endpoints in regulatory contexts.

The following year, RaDaR published outcome data from approximately 28 000 participants across 28 rare kidney disease categories, comparing rates of mortality, kidney function decline and kidney failure with both the general UK population and the 2.8 million UK residents known to have CKD [7]. In addition to documenting the natural history of these conditions, the study revealed striking differences in prognosis: when stratified by age, RaDaR participants with CKD stages 3–5 had a remarkable 28-fold higher 5-year cumulative hazard of kidney failure compared with individuals with CKD stages 3–5 in the general population, but a 2.5-fold lower mortality rate. Among patients receiving dialysis, mortality was also lower among those with rare kidney diseases compared with those with diabetes mellitus or renovascular disease/hypertension as primary diagnoses. These findings help explain the disproportionate contribution of rare kidney diseases to the overall burden of kidney failure, and highlight a critical gap in public health strategy: that further reductions in kidney failure prevalence will require not just incremental improvements in common CKD management, but also the development, and availability to patients, of highly effective therapies specifically targeting rare kidney diseases.

In parallel, a second study, also published in 2024, revealed direct evidence of the socioeconomic consequence of rare kidney disease, particularly for affected children and their families [6], providing additional evidence of the societal burden, beyond healthcare costs, morbidity and mortality, of rare kidney diseases.

A key barrier to development of effective treatments is lack of a clear pathway from drug development to regulatory approval. Such approval requires confidence that the benefits of a therapy (specifically how it affects how patients feel, function or survive) outweigh its known or potential risks. As outlined above, demonstrating this in rare diseases is especially challenging due to the small number of patients, long disease course, and the need to rely on surrogate outcomes such as changes in eGFR or proteinuria. A major hurdle is establishing and quantifying the extent to which these surrogate outcomes reliably predict long-term, clinically meaningful endpoints such as kidney failure or death.

Building on the precedent in IgA nephropathy, recent analyses of RaDaR data in C3 glomerulopathy (C3G) and immune complex membranoproliferative glomerulonephritis (IC-MPGN) have helped address this challenge. These studies showed that while eGFR slope is associated with long-term risk of kidney failure, proteinuria levels at 1 year after diagnosis were a far stronger predictor. For example, patients with proteinuria levels below approximately 1 g/day at 1 year had a 90% lower 20-year risk of kidney failure compared with those above this threshold [13]. These findings directly informed regulatory decision-making by the US Food and Drug Administration [14], which has recently approved two therapies for C3G and/or IC-MPGN in 2025 based on trials that used proteinuria reduction at 6 months as their primary endpoint.

Similarly, analyses conducted by the international PARASOL consortium of registries confirmed that proteinuria reduction (short of complete remission) is strongly associated with a lower risk of kidney failure over 5 years among patients with either primary or genetic focal segmental glomerulosclerosis (FSGS). In contrast, eGFR slope was again a weaker predictor of outcomes in this setting [15]. These findings were consistent in RaDaR analyses [16] and have supported regulatory applications for marketing authorization by at least two companies (Dimerix and Travere Therapeutics) for therapies targeting FSGS, based on trials showing proteinuria reduction as a key efficacy endpoint. Importantly, power analyses performed by the PARASOL group showed that due to the high intra-individual variability in eGFR among patients with FSGS, trials would need to be unfeasibly large to reliably detect moderate treatment effects on eGFR within realistic timeframes. This may help explain the inconclusive eGFR findings in the DUPLEX trial of sparsentan in FSGS, despite a clear effect on proteinuria [17].

Following similar advances in therapeutic development for IgA nephropathy—where data supporting accelerated approval of new therapies based on proteinuria reduction have been published, with confirmatory approval contingent on longer-term effects on eGFR slope [18]—there is justified optimism that the adoption of similarly accessible endpoints in other kidney diseases could further accelerate therapeutic progress.

Nonetheless, one question that RaDaR data alone cannot answer for new drugs is the extent to which a given therapy’s impact on a surrogate endpoint will truly reflect its effect on the hard outcome of kidney failure. In the absence of extended follow-up from clinical trials, such judgements must currently be based on a detailed understanding of the drug’s mechanism of action, integrating biological plausibility with the available clinical and trial evidence to reach a reasoned conclusion for each individual therapy. This underscores the importance of combining registry-based evidence with long-term outcome data and mechanistic insight—derived from both preclinical and clinical studies—to ensure that benefits observed in surrogate endpoints are not only statistically significant, but also translate into meaningful and durable improvements in patient health.

### Clinical trial feasibility and recruitment

RaDaR incorporates near real-time updates of clinical parameters such as eGFR and proteinuria, alongside demographic, diagnostic and histological data (including kidney biopsy reports). This enables the identification of patients who may meet specific clinical trial eligibility criteria, and allows estimation of the number and geographic distribution of potentially eligible participants. Such analyses can be used to assess the feasibility of proposed inclusion criteria and to identify trial sites with the greatest recruitment potential.

Importantly, participation in RaDaR includes consent to be recontacted for research purposes, enabling proactive patient engagement in clinical studies. Where recruitment is especially challenging, RaDaR can connect eligible patients with details of open trial sites, facilitating decentralized and patient-led trial enrolment. This approach has been successfully employed across multiple trials, including in FSGS [19] and Alport syndrome [20], with one such initiative allowing recruitment of very rare patients with nonsense mutations and well-preserved kidney function for a trial of a novel ribosome-targeted readthrough agent—a trial that would very likely have failed to recruit without this infrastructure.

### Economic and health technology appraisals

In the UK, NICE the National Institute for Health and Care Excellence (NICE) is responsible for appraising the use of medicines within the NHS. As is well known to nephrologists and affected patients, modelling the impacts and outcomes in rare kidney diseases using data available from large cohorts of patients with CKD is imperfect. Recent NICE technology appraisals, including TA1074 (sparsentan) and TA937 (targeted-release budesonide), used RaDaR data to validate proteinuria reduction as a surrogate endpoint, model CKD stage transitions and assess the representativeness of trial populations. In the appraisal of HST25 (lumasiran) for primary hyperoxaluria type 1, RaDaR data helped estimate the likely UK patient population eligible for treatment. Looking ahead, ongoing work is leveraging newly established data linkages to HES to more comprehensively capture the economic, healthcare utilization, and broader health impacts of rare kidney diseases and their therapies across the NHS.

### Patient-reported experience measures

RaDaR maintains contact with participants, primarily via e-mail, and has the capability to distribute questionnaires to capture patient-reported experience measures. In response to an initial signal emerging from patient-led social media posts, researchers used RaDaR to conduct a structured investigation into loin pain—first in individuals with IgA nephropathy [21] and subsequently across the broader RaDaR population [22]. Although analyses are ongoing, the study achieved a response rate of almost 25%, yielding more than 3800 completed questionnaires. These early results highlight the significant potential for RaDaR to support the development and validation of tools designed to quantify the real-world impact of rare kidney diseases from the patient perspective.

## FUTURE OUTLOOK

As advances in genomics and molecular medicine deepen our understanding of the biological mechanisms underlying rare kidney diseases, the development of targeted therapies—directed at DNA, mRNA, ribosomes, proteins or cells—is likely to accelerate. However, translating these innovations into treatments accessible to patients remains a significant challenge. Robust, real-world patient datasets have emerged as a valuable tool that can be used to address many of the barriers to therapeutic development, particularly in supporting clinical trials, regulatory approvals and health technology assessments. Current work aims to link RaDaR data with genomic data from UK clinical and research genomic medicine initiatives (such as the 100 000 Genomes Project and NHS Genomic Medicine Service) and it is hoped that analysis of the resulting datasets will yield further insights—perhaps into genomic contributors to outcomes or severity that have not been detected by previous case–control studies.

RaDaR has demonstrated the value of national-scale registry infrastructure to support therapeutic development across multiple domains, including genomic and natural history studies, surrogate endpoint validation, clinical trial design and recruitment, and economic modelling. Yet for the rarest conditions, even national registry datasets may be underpowered. In such cases, international collaboration—through aligned protocols, harmonized endpoints, and federated- or meta-analyses, as exemplified by initiatives such as the PARASOL and ADPedKD [23] consortia—will enable adequately powered studies capable of catalysing change by informing both regulatory and clinical decision-making.

Continued investment in coordinated data systems, training of clinical and statistical researchers, long-term patient engagement, and transnational partnerships will be critical to ensuring that emerging therapeutic advances can be effectively translated into benefit for all those living with rare kidney diseases.
